# Locoregional Extension Patterns of Nasopharyngeal Carcinoma Detected by FDG PET/MR

**DOI:** 10.3389/fonc.2021.763114

**Published:** 2021-12-13

**Authors:** Caineng Cao, Yuanfan Xu, Shuang Huang, Feng Jiang, Ting Jin, Qifeng Jin, Yonghong Hua, Qiaoying Hu, Xiaozhong Chen

**Affiliations:** ^1^ Department of Radiation Oncology, Cancer Hospital of the University of Chinese Academy of Sciences (Zhejiang Cancer Hospital), Institute of Cancer Research and Basic Medicine (ICBM), Chinese Academy of Sciences, Zhejiang Provincial Key Laboratory of Radiation Oncology, Hangzhou, China; ^2^ Key Laboratory of Head and Neck Cancer Translational Research of Zhejiang Province, Hangzhou, China; ^3^ Hangzhou Universal Medical Imaging Diagnostic Center, Hangzhou, China

**Keywords:** nasopharyngeal carcinoma, simultaneous PET/MRI, clinical target volume, local extension, lymph node spread

## Abstract

**Purpose:**

We sought to define the locoregional extension patterns of nasopharyngeal carcinomas (NPCs) by positron emission tomography (PET)/magnetic resonance imaging (MRI) and to improve clinical target volume (CTV) delineation.

**Methods:**

Between May 2017 and March 2021, 331 consecutive patients with nonmetastatic NPCs who underwent pretreatment, simultaneous whole-body PET/MRI for staging were included in this study.

**Results:**

The high-risk regions included the base of the sphenoid bone, the prestyloid compartment, prevertebral muscle, foramen lacerum, medial pterygoid plate, sphenoidal sinus, clivus, petrous apex, and foramen ovale. When the high-risk regions were invaded, the incidence rates of tumor invasion into the medium-risk regions increased. In contrast, when the high-risk regions were not involved, the incidence rates of tumor invasion into the medium-risk regions were mostly less than 10%, excluding the post-styloid compartment and oropharynx. According to the updated consensus guidelines of the neck node levels for head and neck tumors from 2013, level IIa (77.3%, 256/331), level IIb (75.8%, 251/331), and level VIIa (71.3%, 236/331) were the most frequently involved levels, followed by levels III (42.6%), Va (13.9%), IVa (8.8%), IVb (3.6%), Ib (3.6%), Vb (2.4%), VIIb (2.4%), VIII (1.8%), Vc (0.9%), and Xa (0.3%). Skip lymph node metastasis occurred in only 1.9% of patients.

**Conclusions:**

For NPCs, primary disease and regional lymph node spread follow an orderly pattern, and a skip pattern of lymph node metastasis was unusual. Involved level radiotherapy might be feasible for cervical lymph node levels below the caudal border of cricoid cartilage and level VIIb.

## Introduction

Radiotherapy is the primary treatment modality for nonmetastatic nasopharyngeal carcinomas (NPCs) (1). Target delineation of NPCs is often challenging for the proximity of the tumors to critical organs at risk such as the brain stem and spinal cord. Based on the best available investigation methods, accurate delineation of the Gross Tumor Volume (GTV) is the first step. The next step is to delineate the clinical target volume (CTV) covering the subclinical microscopic malignant lesions. However, there are many controversies on details of contouring in NPCs (2).

In the National Comprehensive Cancer Network (NCCN) guidelines, magnetic resonance imaging (MRI) with a contrast of skull base to clavicle is recommended for defining the locoregional extension of NPC, and 18 fluorodeoxyglucose (FDG) positron emission tomography (PET)/computed tomography (CT) is merely recommended for nodal and distant metastases in patients with multistation or lower neck nodal involvement or high-grade tumor histology (3). The diagnostic criteria of lymph node involvement included lymph nodes with overt FDG uptake in the international guideline for target volume delineation of NPC (4). A prospective study indicated that PET/MRI was more accurate than the combination of head and neck MRI and PET/CT in the staging of newly diagnosed NPCs (5). With the development of PET/MR, the target volume delineation of NPCs should be reevaluated.

In our previous studies, PET/MRI for dose painting and staging was evaluated in NPC (6, 7). The aim of the present study was to define the locoregional extension patterns of NPC by PET/MRI and to improve CTV delineation.

## Materials and Methods

### Patients

Between May 2017 and March 2021, 331 consecutive patients with biopsy-proven, newly diagnosed, nonmetastatic NPCs were included in this study. The pretreatment evaluation included a complete history and physical examination, fiber-optic nasopharyngoscopy, MRI of the nasopharynx and neck, and whole-body 18F-FDG PET/MR. Medical records and imaging studies were analyzed retrospectively, and all patients were staged according to the 8th edition of the American Joint Committee on Cancer (AJCC)/Union for International Cancer Control (UICC) staging system (8, 9). Patient clinicopathologic characteristics are listed in **Table 1**.

**Table 1:** Patient characteristics.

Characteristic	No. (%)
Sex	
Male	254 (76.7)
Female	77 (23.3)
Age (years)	
Median	51
Range	13–81
Pathology classification	
Non-keratinizing	331 (100.0)
T classification	
T1	22 (6.6)
T2	25 (7.6)
T3	189 (57.1)
T4	95 (28.7)
N classification	
N0	20 (6.0)
N1	81 (24.5)
N2	169 (51.1)
N3	61 (18.4)
Overall stage	
I	3 (0.9)
II	7 (2.1)
III	179 (54.1)
IVA	142 (42.9)

Data in parentheses are percentages.

### Whole-Body ^18^F-FDG PET/MR

PET/MRI was performed on a SIGNA PET/MR (GE Healthcare) by a 3-T magnetic field strength, total imaging matrix coil technology, and a fully functional PET system. The scan range covered the entire body. The details of PET/MR were described previously (6).

### Image Interpretation and Diagnostic Criteria

Interpretation of the PET/MRI fused images, plus their individual MRI and PET components, was evaluated independently by two nuclear medicine physicians who also had qualification certificates in radiology. Any discrepancy was resolved by consensus. In addition, there is a multidisciplinary team of NPC to confirm the extent of diseases and the treatment in our center (10).

Nasal cavity invasion was defined as a primary tumor invading beyond the posterior line of the pterygopalatine fossa. Oropharynx invasion was defined as a primary tumor invading below C1/C2. Hypopharynx invasion was defined as a primary tumor invading below or the free edge of the epiglottis or the lower margin of C3. The parapharyngeal space included the prestyloid and poststyloid compartments. Parapharyngeal space invasion was defined as primary tumor invading posterolaterally beyond the pharyngobasilar fascia. Infratemporal fossa invasion was defined as a primary tumor invading beyond the posterolateral wall of the maxillary sinus or pterygomaxillary fissure or the anterior surface of the lateral pterygoid muscle (11). Paranasal sinuses invasion was defined as a primary tumor invading into the sinus cavity with bone destruction of the wall of the sinus (10). Cavernous sinus invasion was defined as a change in contour or enlargement of the sinus (12). The primary tumor invading across the midline of the nasopharynx was classified as bilateral NPC excluding the midline anatomic sites, such as the clivus and base of the sphenoid bone. Based on the incidence rates of primary tumor involvement, the regions surrounding the nasopharynx were divided into high-risk (≥35%), medium-risk (≥5%–35%), and low-risk (<5%) regions (11).

The diagnostic criteria of regional lymph node metastases included (a) lateral retropharyngeal nodes with minimal axial diameter in the largest plane > 5 mm (any node seen in the median retropharyngeal group) or cervical lymph nodes with minimal axial diameter in the largest plane > 10 mm (11 mm for subdigastric lymph nodes); (b) three or more contiguous and confluent lymph nodes, each with the shortest diameter of 8–10 mm; and (c) lymph nodes of any size with extracapsular spread, central necrosis, or overt FDG uptake (4, 7). The assignment of neck node levels was made with reference to the 2013 updated consensus guidelines of the neck node levels for head and neck tumors (13).

### Statistical Analysis

The Statistical Package for Social Sciences, version 17.0 (SPSS Inc., Chicago, IL), software was used for statistical analysis. The Chi-squared and Fisher’s exact tests were used to compare the differences between categorical variables, and a *p* < 0.05 was considered to be statistically significant.

## Results

### The Risk of Tumor Invasion Into Various Anatomic Sites

The incidence rates of tumor invasion into anatomic sites for NPCs are shown in **Table 2**. The high-risk regions included the basis of the sphenoid bone, prestyloid compartment, prevertebral muscle, foramen lacerum, medial pterygoid plate, sphenoidal sinus, clivus, petrous apex, and foramen ovale. Excluding the nasal cavity and oropharynx, the high-risk regions were adjacent to the nasopharynx. The medium-risk regions included meninges, pterygopalatine fossa, poststyloid compartment, cavernous sinus, medial pterygoid muscle, oropharynx, hypoglossal canal, foramen rotundum, lateral pterygoid plate, jugular foramen, nasal cavity, parotid gland, and lateral pterygoid muscle.

**Table 2:** Incidence rates of tumor invasion into anatomic sites for nasopharyngeal carcinoma.

Anatomic sites	No. of patients (%)
High risk	
Basis of sphenoid bone	271 (81.9)
Prestyloid compartment	234 (70.7)
Prevertebral muscle	182 (55.0)
Foramen lacerum	176 (53.2)
Medial pterygoid plate	160 (48.3)
Sphenoidal sinus	153 (46.2)
Clivus	150 (45.3)
Petrous apex	135 (40.8)
Foramen ovale	122 (36.9)
Medium risk	
Meninges	91 (27.5)
Pterygopalatine fossa	80 (24.2)
Poststyloid compartment	80 (24.2)
Cavernous sinus	75 (22.7)
Medial pterygoid muscle	69 (20.8)
Oropharynx	58 (17.5)
Hypoglossal canal	54 (16.3)
Foramen rotundum	52 (15.7)
Lateral pterygoid plate	47 (14.2)
Jugular foramen	37 (11.2)
Nasal cavity	25 (7.6)
Parotid gland	21 (6.3)
Lateral pterygoid muscle	19 (5.7)
Low risk	
Orbit	13 (3.9)
Ethmoid sinus	13 (3.9)
Maxillary sinus	12 (3.6)
Infratemporal fossa	5 (1.5)
Temporal lobe	1 (0.3)
Hypopharynx	1 (0.3)

When the high-risk regions were invaded, the incidence rates of tumor invasion into the medium-risk regions increased. In the case of the tumor invading the foramen ovale, 64.8% of patients developed meninges involvement. In contrast, when the high-risk regions were not involved, the incidence rates of tumor invasion into the medium-risk regions were mostly less than 10% excluding the poststyloid compartment and oropharynx (**Table 3**).

**Table 3:** Relationship between primary tumor invasion into the high-risk regions and the medium risk regions.

Medium-risk regions	Tumor invasion		*p*
	**Basis of sphenoid bone**		
	**Invasion (*n* = 271)**	**Non-invasion (*n* = 60)**	
Meninges	91 (33.6%)	0 (0.0%)	<0.001
Pterygopalatine fossa	79 (29.2%)	1 (1.7%)	<0.001
Poststyloid compartment	73 (26.9%)	7 (11.7%)	0.012
Cavernous sinus	75 (27.7%)	0 (0.0%)	<0.001
Medial pterygoid muscle	66 (24.4%)	3 (5.0%)	<0.001
Oropharynx	51 (18.8%)	7 (11.7%)	0.187
Hypoglossal canal	53 (19.6%)	1 (1.7%)	<0.001
Foramen rotundum	52 (19.2)	0 (0.0%)	<0.001
Lateral pterygoid plate	47 (17.3%)	0 (0.0%)	<0.001
Jugular foramen	36 (13.3%)	1 (1.7%)	0.006
Nasal cavity	25 (9.2%)	0 (0.0%)	0.012
Parotid gland	20 (7.4%)	1 (1.7%)	0.142
Lateral pterygoid muscle	19 (7.0%)	0 (0.0%)	0.031
	**Prestyloid compartment**		
	**Invasion (*n* = 234)**	**Non-invasion (*n* = 97)**	
Meninges	83 (35.5%)	8 (8.2%)	<0.001
Pterygopalatine fossa	74 (31.6%)	6 (6.2%)	<0.001
Poststyloid compartment	80 (34.2%)	0 (0.0%)	<0.001
Cavernous sinus	71 (30.3%)	4 (4.1%)	<0.001
Medial pterygoid muscle	69 (29.5%)	0 (0.0%)	<0.001
Oropharynx	58 (24.8%)	0 (0.0%)	<0.001
Hypoglossal canal	51 (21.8%)	3 (3.1%)	<0.001
Foramen rotundum	49 (20.9%)	3 (3.1%)	<0.001
Lateral pterygoid plate	47 (20.1%)	0 (0.0%)	<0.001
Jugular foramen	35 (15.0%)	2 (2.1%)	<0.001
Nasal cavity	21 (9.0%)	4 (4.1%)	0.171
Parotid gland	21 (9.0%)	0 (0.0%)	0.001
Lateral pterygoid muscle	19 (8.1%)	0 (0.0%)	0.001
	**Prevertebral muscle**		
	**Invasion (*n* = 182)**	**Non-invasion (*n* = 149)**	
Meninges	82 (45.1%)	9 (6.0%)	<0.001
Pterygopalatine fossa	59 (32.4%)	21 (14.1%)	<0.001
Poststyloid compartment	78 (42.9%)	2 (1.3%)	<0.001
Cavernous sinus	68 (37.4%)	7 (4.7%)	<0.001
Medial pterygoid muscle	56 (30.8%)	13 (8.7%)	<0.001
Oropharynx	51 (28.0%)	7 (4.7%)	<0.001
Hypoglossal canal	54 (29.7%)	0 (0.0%)	<0.001
Foramen rotundum	44 (24.2%)	8 (5.4%)	<0.001
Lateral pterygoid plate	37 (20.3%)	10 (6.7%)	<0.001
Jugular foramen	36 (19.8%)	1 (0.7%)	<0.001
Nasal cavity	13 (7.1%)	12 (8.1%)	0.755
Parotid gland	21 (11.5%)	0 (0.0%)	<0.001
Lateral pterygoid muscle	15 (8.2%)	4 (2.7%)	0.034
	**Foramen lacerum**		
	**Invasion (*n* = 176)**	**Non-invasion (*n* = 155)**	
Meninges	90 (51.1%)	1 (0.6%)	<0.001
Pterygopalatine fossa	69 (39.2%)	11 (7.1%)	<0.001
Poststyloid compartment	71 (40.3%)	9 (5.8%)	<0.001
Cavernous sinus	74 (42.0%)	1 (0.6%)	<0.001
Medial pterygoid muscle	64 (36.4%)	5 (3.2%)	<0.001
Oropharynx	49 (27.8%)	9 (5.8%)	<0.001
Hypoglossal canal	54 (30.7%)	0 (0.0%)	<0.001
Foramen rotundum	49 (27.8%)	3 (1.9%)	<0.001
Lateral pterygoid plate	44 (25.0%)	3 (1.9%)	<0.001
Jugular foramen	37 (21.0%)	0 (0.0%)	<0.001
Nasal cavity	19 (10.8%)	6 (3.9%)	0.017
Parotid gland	21 (11.9%)	0 (0.0%)	<0.001
Lateral pterygoid muscle	19 (10.8%)	0 (0.0%)	<0.001
	**Medial pterygoid plate**		
	**Invasion (*n* = 160)**	**Non-invasion (*n* = 171)**	
Meninges	75 (46.9%)	16 (9.4%)	<0.001
Pterygopalatine fossa	78 (48.8%)	2 (1.2%)	<0.001
Poststyloid compartment	57 (35.6%)	23 (13.5%)	<0.001
Cavernous sinus	61 (38.1%)	14 (8.2%)	<0.001
Medial pterygoid muscle	67 (41.9%)	2 (1.2%)	<0.001
Oropharynx	42 (26.3%)	16 (9.4%)	<0.001
Hypoglossal canal	41 (25.6%)	13 (7.6%)	<0.001
Foramen rotundum	48 (30.0%)	4 (2.3%)	<0.001
Lateral pterygoid plate	47 (29.4%)	0 (0.0%)	<0.001
Jugular foramen	29 (18.1%)	8 (4.7%)	<0.001
Nasal cavity	24 (15.0%)	1 (0.6%)	<0.001
Parotid gland	19 (11.9%)	2 (1.2%)	<0.001
Lateral pterygoid muscle	19 (11.9%)	0 (0.0%)	<0.001
	**Sphenoidal sinus**		
	**Invasion (*n* = 153)**	**Non-invasion (*n* = 178)**	
Meninges	91 (59.5%)	0 (0.0%)	<0.001
Pterygopalatine fossa	69 (45.1%)	11 (6.2%)	<0.001
Poststyloid compartment	59 (38.6%)	21 (11.8%)	<0.001
Cavernous sinus	75 (49.0%)	0 (0.0%)	<0.001
Medial pterygoid muscle	61 (39.9%)	8 (4.5%)	<0.001
Oropharynx	39 (25.5%)	19 (10.7%)	<0.001
Hypoglossal canal	51 (33.3%)	3 (1.7%)	<0.001
Foramen rotundum	52 (34.0%)	0 (0.0%)	<0.001
Lateral pterygoid plate	45 (29.4%)	2 (1.1%)	<0.001
Jugular foramen	34 (22.2%)	3 (1.7%)	<0.001
Nasal cavity	21 (13.7%)	4 (2.2%)	<0.001
Parotid gland	20 (13.1%)	1 (0.6%)	<0.001
Lateral pterygoid muscle	19 (12.4%)	0 (0.0%)	<0.001
	**Clivus**		
	**Invasion (*n* = 150)**	**Non-invasion (*n* = 181)**	
Meninges	83 (55.3%)	8 (4.4%)	<0.001
Pterygopalatine fossa	59 (39.3%)	21 (11.6%)	<0.001
Poststyloid compartment	64 (42.7%)	16 (8.8%)	<0.001
Cavernous sinus	69 (46.0%)	6 (3.3%)	<0.001
Medial pterygoid muscle	58 (38.7%)	11 (6.1%)	<0.001
Oropharynx	43 (28.7%)	15 (8.3%)	<0.001
Hypoglossal canal	53 (35.3%)	1 (0.6%)	<0.001
Foramen rotundum	43 (28.7%)	9 (5.0%)	<0.001
Lateral pterygoid plate	40 (26.7%)	7 (3.9%)	<0.001
Jugular foramen	36 (24.0%)	1 (0.6%)	<0.001
Nasal cavity	15 (10.0%)	10 (5.5%)	0.125
Parotid gland	20 (13.3%)	1 (0.6%)	<0.001
Lateral pterygoid muscle	15 (10.0%)	4 (2.2%)	0.003
	**Petrous apex**		
	**Invasion (*n* = 135)**	**Non-invasion (*n* = 196)**	
Meninges	85 (63.0%)	6 (3.1%)	<0.001
Pterygopalatine fossa	56 (41.5%)	24 (12.2%)	<0.001
Poststyloid compartment	68 (50.4%)	12 (6.1%)	<0.001
Cavernous sinus	70 (51.9%)	5 (2.6%)	<0.001
Medial pterygoid muscle	58 (43.0%)	11 (5.6%)	<0.001
Oropharynx	46 (34.1%)	12 (6.1%)	<0.001
Hypoglossal canal	54 (40.0%)	0 (0.0%)	<0.001
Foramen rotundum	43 (31.9%)	9 (4.6%)	<0.001
Lateral pterygoid plate	40 (29.6%)	7 (3.6%)	<0.001
Jugular foramen	37 (27.4%)	0 (0.0%)	<0.001
Nasal cavity	12 (8.9%)	13 (6.6%)	0.445
Parotid gland	21 (15.6%)	0 (0.0%)	<0.001
Lateral pterygoid muscle	17 (12.6%)	2 (1.0%)	<0.001
	**Foramen ovale**		
	**Invasion (*n* = 122)**	**Non-invasion (*n* = 209)**	
Meninges	79 (64.8%)	12 (5.7%)	<0.001
Pterygopalatine fossa	56 (45.9%)	24 (11.5%)	<0.001
Poststyloid compartment	63 (51.6%)	17 (8.1%)	<0.001
Cavernous sinus	66 (54.1%)	9 (4.3%)	<0.001
Medial pterygoid muscle	55 (45.1%)	14 (6.7%)	<0.001
Oropharynx	39 (32.0%)	19 (9.1%)	<0.001
Hypoglossal canal	49 (40.2%)	5 (2.4%)	<0.001
Foramen rotundum	45 (36.9%)	7 (3.3%)	<0.001
Lateral pterygoid plate	39 (32.0%)	8 (3.8%)	<0.001
Jugular foramen	35 (28.7%)	2 (1.0%)	<0.001
Nasal cavity	15 (12.3%)	10 (4.8%)	0.013
Parotid gland	19 (15.6%)	2 (1.0%)	<0.001
Lateral pterygoid muscle	17 (13.9%)	2 (1.0%)	<0.001

### Tumor Invasion Into Bilateral Anatomical Sites

Three hundred and nineteen patients (319/331, 96.4%) had bilateral tumor invasion into the mucous membrane of the nasopharynx. The details of concurrent bilateral tumor invasion into anatomic sites for NPC are shown in **Table 4**. Of note, the incidence rates of concurrent bilateral tumor invasion were mostly less than 10%, excluding the prevertebral muscle (16.3%).

**Table 4:** Concurrent bilateral tumor invasion into anatomic sites for nasopharyngeal carcinoma.

Anatomic sites	No. of patients (%)
Prevertebral muscle	54 (16.3)
Foramen lacerum	32 (9.7)
Prestyloid compartment	28 (8.5)
Petrous apex	14 (4.2)
Medial pterygoid plate	12 (3.6)
Foramen ovale	7 (2.1)
Hypoglossal canal	6 (1.8)
Pterygopalatine fossa	5 (1.5)
Foramen rotundum	3 (0.9)
Medial pterygoid muscle	3 (0.9)
Poststyloid compartment	3 (0.9)
Oropharynx	3 (0.9)
Nasal cavity	2 (0.6)
Jugular foramen	2 (0.6)
Orbit	1 (0.3)
Maxillary sinus	0 (0)
Lateral pterygoid plate	0 (0)
Lateral pterygoid muscle	0 (0)

### Skull-Base Invasion

The cumulative rates of tumor invasion into the anatomic sites of the skull are shown in **Table 5**. The base of sphenoid bone had the highest involvement rate (81.9%), followed by the foramen lacerum (53.2%) and medial pterygoid plate (48.3%). The incidence rates of tumor invasion into the bilateral anatomic sites such as foramen lacerum, petrous apex, medial pterygoid plate, and foramen ovale were less than 10%. No significant difference was observed in terms of cumulative incidence rates between right and left anatomic sites of skull-base invasion.

**Table 5:** Incidence rates of skull-base invasion for nasopharyngeal carcinoma.

Site of skull-base invasion	Bilateral	Left	Right
Base of sphenoid bone	271 (81.9)		
Clivus	150 (45.3)		
Foramen lacerum	32 (9.7)	70 (21.1)	74 (22.4)
Petrous apex	14 (4.2)	62 (18.7)	59 (17.8)
Medial pterygoid plate	12 (3.6)	81 (24.5)	67 (20.2)
Foramen ovale	7 (2.1)	54 (16.3)	61 (18.4)
Pterygopalatine fossa	5 (1.5)	36 (10.9)	39 (11.8)
Foramen rotundum	3 (0.9)	22 (6.6)	27 (8.2)
Hypoglossal canal	6 (1.8)	24 (7.3)	24 (7.3)
Lateral pterygoid plate	0 (0)	20 (6.0)	27 (8.2)
Jugular foramen	2 (0.6)	17 (5.1)	18 (5.4)

### Pattern of Regional Lymph Node Metastasis

**Figure 1:**
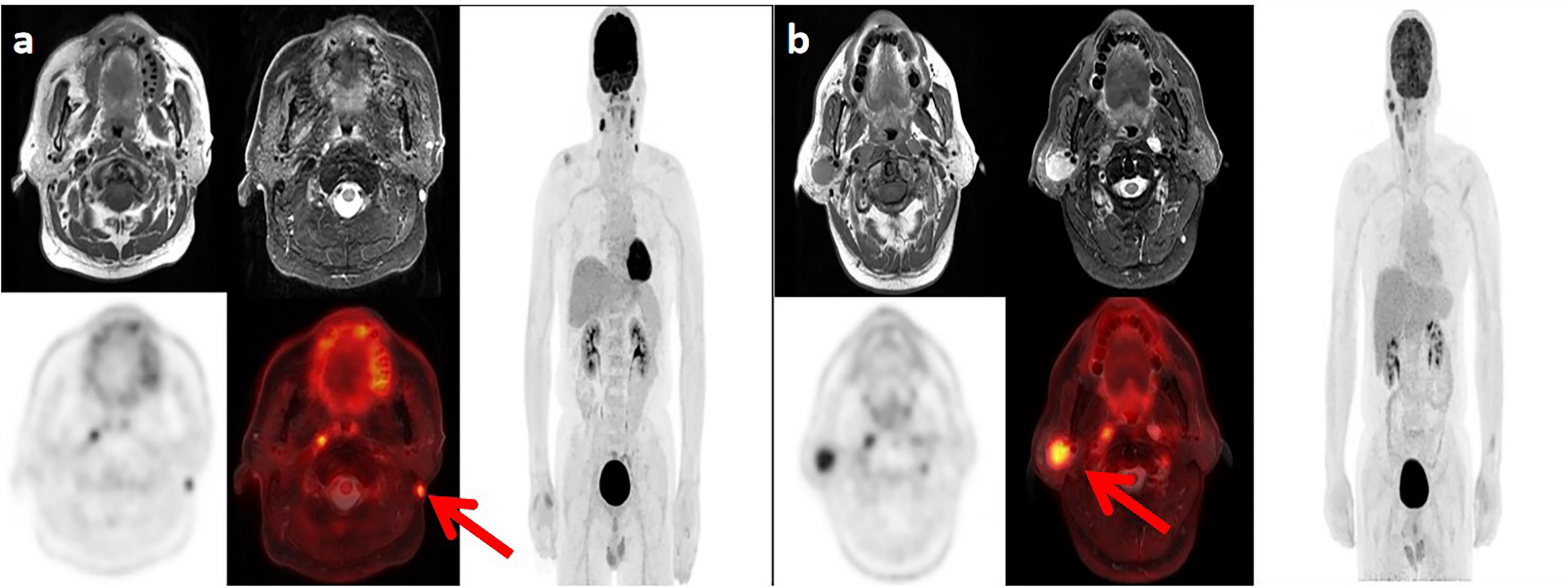
Lymph node metastases in two patients with nasopharyngeal carcinoma. **(A)** Positron emission tomography (PET)/magnetic resonance imaging (MRI) in a 62-year-old man show the left level Xa lymph node metastasis. **(B)** PET/MRI in a 47-year-old man show the right level VIII lymph node metastasis.

In this cohort of patients, the rate of lymph node metastasis was 94.0% (311/331), and bilateral lymph node metastasis was observed in 74.9% (248/331) of patients. With reference to 2013 updated consensus guidelines of the neck node levels for head and neck tumors, level IIa (77.3%, 256/331), level IIb (75.8%, 251/331), and level VIIa (71.3%, 236/331) were the most frequently involved levels, followed by levels III (42.6%, 141/331), Va (13.9%, 46/331), IVa (8.8%, 29/331), IVb (3.6%, 12/331), Ib (3.6%, 12/331), Vb (2.4%, 8/331), VIIb (2.4%, 8/331), VIII (1.8%, 6/331), Vc (0.9%, 3/331), and Xa (0.3%, 1/331) (**Figure 1**). One patient showed a medial group of retropharyngeal lymph node metastasis. No lymph node metastasis was observed in levels Ia, VI, IX, or Xb (**Table 6**).

**Table 6:** Incidence rates of nodal spread for nasopharyngeal carcinoma.

Level	Bilateral	Left	Right
Level VIIa*	98 (29.6)	62 (18.7)	76 (23.0)
Level VIIb	0 (0)	5 (1.5)	3 (0.9)
Level IIa	149 (45.0)	52 (15.7)	55 (16.6)
Level IIb	147 (44.4)	51 (15.4)	53 (16.0)
Level III	32 (9.7)	59 (17.8)	50 (15.1)
Level Va	2 (0.6)	23 (6.9)	21 (6.3)
Level IVa	2 (0.6)	14 (4.2)	13 (3.9)
Level IVb	1 (0.3)	8 (2.4)	3 (0.9)
Level Vb	0 (0)	6 (1.8)	2 (0.6)
Level Vc	0 (0)	2 (0.6)	1 (0.3)
Level Ib	2 (0.6)	7 (2.1)	3 (0.9)
Level VIII	0 (0)	5 (1.5)	1 (0.3)
Level Xa	0 (0)	1 (0.3)	0 (0)

*Medial group of retropharyngeal lymph node metastasis in 1 patient.

**Figure 2:**
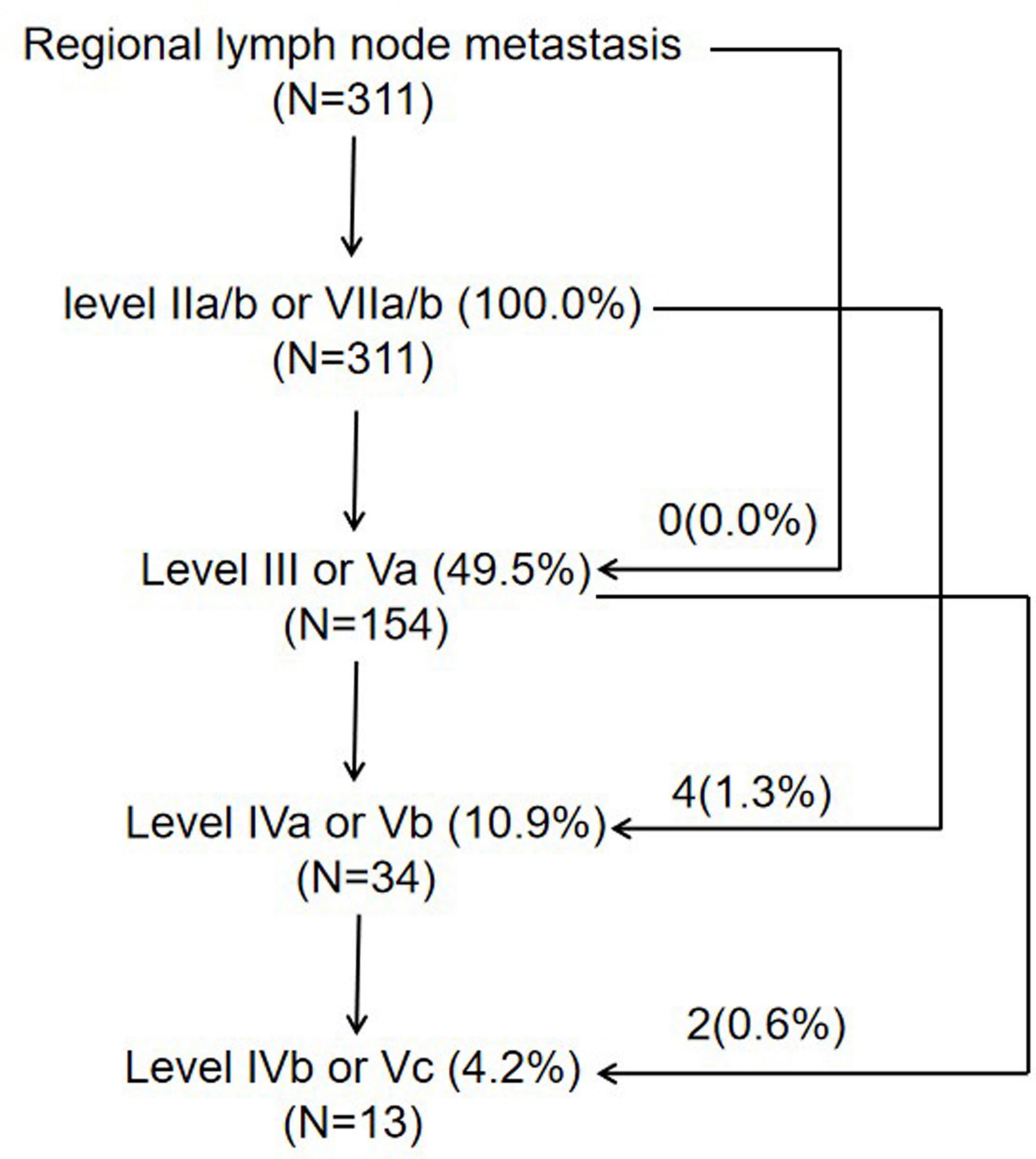
The pattern of lymph node metastasis.

In the 311 patients with lymph node metastasis, the involvement rates of the upper neck (levels IIa, IIb, VIIa, and VIIb), middle neck (levels III and Va), and lower neck (levels IVa, IVb, Vb, and Vc) were 100.0% (311/311), 49.5% (154/311), and 11.6% (36/311), respectively, and skip metastasis occurred in only 1.9% (6/311) of patients (**Figure 2**). In the 64 patients who had unilateral upper neck involvement, the involvement rates of ipsilateral levels III, Va, IVa, IVb, Vb, and Vc were 26.6% (17/64), 12.5% (8/64), 3.1% (2/64), 1.6% (1/64), 3.1% (2/64), and 0.0% (0/64), respectively, whereas the involvement rate of contralateral level III was only 1.6% (1/64) and no lymph node metastasis was observed in contralateral levels Va, IVa, IVb, Vb, and Vc. For patients with bilateral upper neck involvement, the involvement rates of levels III, Va, IVa, IVb, Vb, and Vc were 50.2% (124/247), 15.4% (38/247), 10.9% (27/247), 4.5% (11/247), 2.4% (6/247), and 1.2% (3/247), respectively. 

## Discussion

The emergence of PET/MR has not only brought about higher accuracy for staging patients with NPCs, but it has also reduced radiation dose and wait time for different scans (5). However, the role of PET/MR for the target volume delineation of NPC is limited. This study has shown the locoregional extension patterns based on PET/MRI in NPCs by concrete evidence to improve CTV delineation.

For the local extension pattern, the risk of tumor invasion into various anatomic sites surrounding the nasopharynx was closely associated with the distance to the nasopharynx. The regions at high risk, such as the basis of the sphenoid bone, prestyloid compartment, and prevertebral muscle were adjacent to the nasopharynx, whereas the regions at medium or low risk (except nasal cavity and oropharynx) were distant from the nasopharynx. When the high-risk regions were involved, the rates of tumor invasion into the medium-risk regions increased especially for the adjacent medium-risk regions. Our data indicate that the primary disease spreads stepwise from proximal regions to distal regions and primary disease skipping is unusual, which is similar to the findings of head and neck MRI (11, 14).

King et al. reported 246 patients suspected of having NPC who underwent MRI, endoscopy, and endoscopic biopsy. The sensitivity, specificity, and accuracy of MRI were 100%, 93%, and 95%, respectively (15). In the present study, 94.0% of patients had bilateral tumor invasion in the nasopharynx. However, the regions including the skull base foramina are at low risk of concurrent bilateral tumor invasion (<10%) with the exception of the prevertebral muscle. In the study including 205 NPC patients with intracranial extension, the incidence rates of concurrent bilateral tumor invasion into the foramen lacerum, foramen ovale, and foramen rotundum were 47.8%, 8.8%, and 6.8%, respectively (16). The CTV including the whole nasopharynx and bilateral foramina ovale, foramina rotunda, and foramina lacera irrespective of T classification should be reevaluated (4).

For the pattern of regional lymph node metastasis, the rates of lymph node metastasis from the upper neck to the lower neck decreased successively, and skip metastasis happened only in 1.9% of patients, which further confirms the MRI findings from Li et al. (14). In the Li et al.’s study using head and neck MRI (14), the rates of lymph node metastasis of the middle and lower neck for patients with cervical lymph node metastasis were 30.2% and 7.2%, respectively, which were lower than the present study using PET/MRI. In the study including 113 patients with histologically confirmed NPC, the sensitivity of PET/MRI (99.5%) was higher than that of head and neck MRI (94.2%) in terms of the N staging assessment (5).

**Figure 3:**
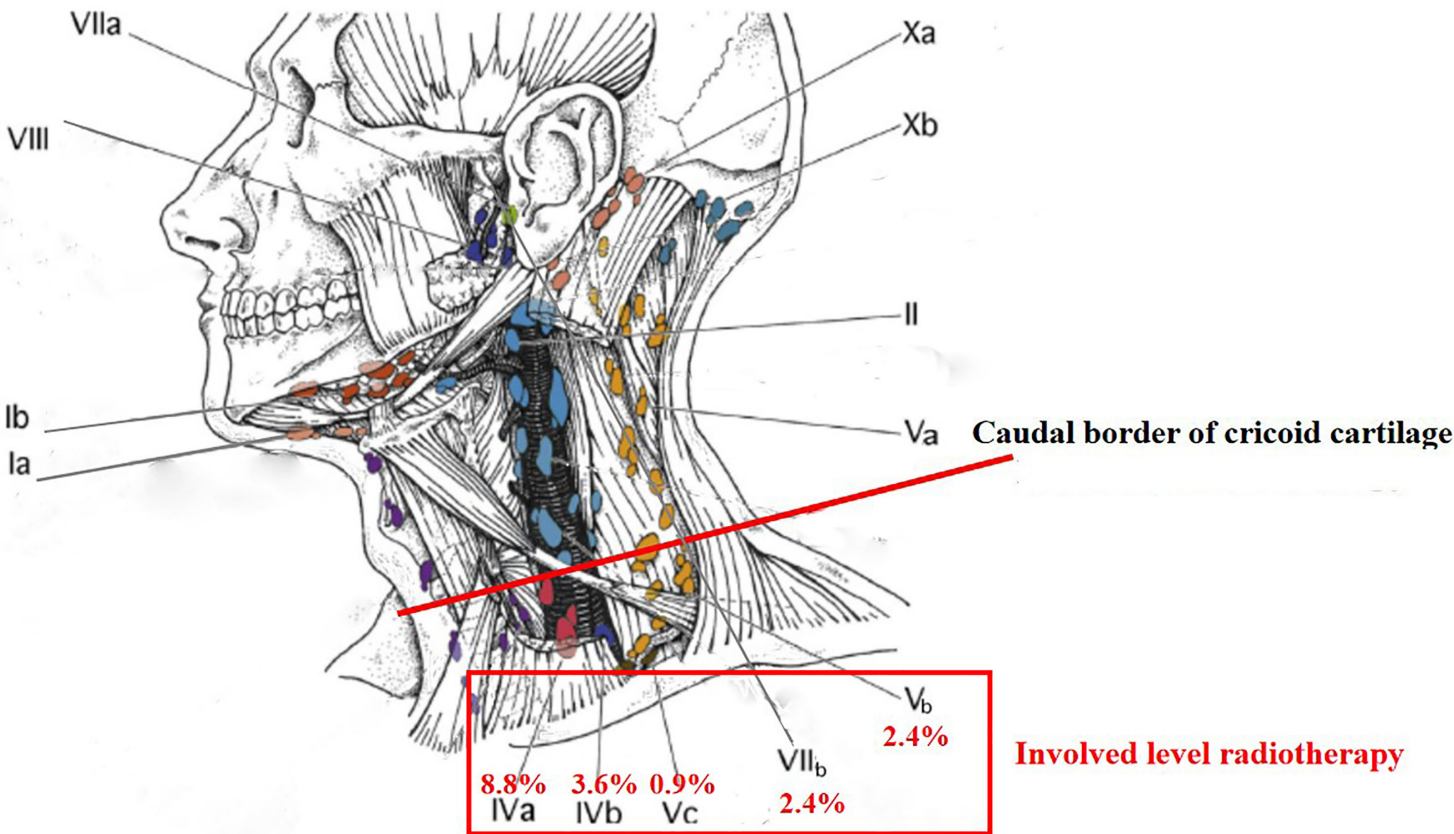
Involved level radiotherapy for nasopharyngeal carcinoma.

According to the 2013 updated consensus guidelines of the neck node levels, the involvement rates of levels IVb, Vb, Vc, and VIIb were not higher than level Ib (3.6%). In the present international guideline for the delineation of the CTV for NPC, level Ib was not included in the CTV, except the special cases, such as involvement of the submandibular gland (4). For patients who had unilateral upper neck involvement, the involvement rates of ipsilateral levels III, Va, IVa, IVb, Vb, and Vc were 26.6%, 12.5%, 3.1%, 1.6%, 3.1%, and 0.0%, respectively, whereas the involvement rate of contralateral level III was only 1.6% and no lymph node metastasis was observed in contralateral levels Va, IVa, IVb, Vb, and Vc. It might be unreasonable to cover cervical lymph node levels IVa, IVb, Vb, and Vc ipsilaterally for CTV if there are any involved lymph nodes in the ipsilateral neck (excluding retropharyngeal lymph nodes) (4, 17). With the development of imaging and treatment, radiotherapy for Hodgkin lymphoma has changed from the very large extended-field radiotherapy to the very limited involved-site radiotherapy (18). For NPC in the era of PET/MR, involved level radiotherapy might be feasible for cervical lymph node levels below the caudal border of the cricoid cartilage and level VIIb (**Figure 3**).

In 2020, Zhang et al. reported the current CTV delineation for the primary site of T3 classification NPC among five large tertiary cancer centers in China. Two different CTV designs were used and the variances in the coverage of some regions including cavernous sinus, posterior space of styloid process, and posterior pharyngeal wall were significant among physicians (19). Guo et al. reported 471 patients with non-metastatic NPCs treated by de-intensification technique omitting the CTV1 and narrowing the margin of CTV2 from 10 mm to 8 mm. The 4-year local recurrence-free survival and overall survival rates were 96.6% and 92.4%, respectively (2). Most commonly, the GTV is homogeneously covered during radiotherapy planning. However, tumors are known to be very heterogeneous. The use of PET/CT to construct a tumor voxel dose-response matrix and dose prescription function for adaptive dose painting by number has been investigated in head and neck cancer (20). With the development of artificial intelligence, automated delineation and segmentation methods have been adopted for NPC and PET/MR radiomics-based dose painting should be evaluated in the near future (21, 22).

## Conclusion

For NPCs, primary disease and regional lymph node spread follow an orderly pattern and a skip pattern of lymph node metastasis was unusual. Involved level radiotherapy might be feasible for cervical lymph node levels below the caudal border of the cricoid cartilage and level VIIb.

## Data Availability Statement

The raw data supporting the conclusions of this article will be made available by the authors, without undue reservation.

## Ethics Statement

The studies involving human participants were reviewed and approved by the Hospital Ethics Committee at Zhejiang Cancer Hospital. Written informed consent for participation was not required for this study in accordance with the national legislation and the institutional requirements.

## Author Contributions

Study conception and design: CC and XC. Data acquisition: CC, YX, SH, FJ, TJ, QJ, YH, QH, and XC. Data analysis and interpretation: YX, SH, FJ, TJ, QJ, YH, and QH. Quality control of data and algorithms: CC, YX, SH, FJ, TJ, QJ, YH, QH, and XC. Manuscript writing: CC and XC. Manuscript reviewing and approving: CC and XC. All authors contributed to the article and approved the submitted version.

## Funding

This work was supported by a grant from the Natural Science Foundation of Zhejiang Province (Nos. LGF19H160007 and LGF20H160007).

## Conflict of Interest

The authors declare that the research was conducted in the absence of any commercial or financial relationships that could be construed as a potential conflict of interest.

## Publisher’s Note

All claims expressed in this article are solely those of the authors and do not necessarily represent those of their affiliated organizations, or those of the publisher, the editors and the reviewers. Any product that may be evaluated in this article, or claim that may be made by its manufacturer, is not guaranteed or endorsed by the publisher.
